# The impact of users’ cognitive function on evaluator perceptions of usability

**DOI:** 10.1038/s41598-022-17441-3

**Published:** 2022-08-12

**Authors:** Ana Isabel Martins, Anabela G. Silva, Joana Pais, Vítor Tedim Cruz, Nelson P. Rocha

**Affiliations:** 1 Center for Health Technology and Services Research-CINTESIS@RISE, University of Aveiro, Aveiro, Portugal; 2 Center for Health Technology and Services Research-CINTESIS@RISE, School of Health Sciences, University of Aveiro, Aveiro, Portugal; 3 EPIUnit - Institute of Public Health, Laboratory for Integrative and Translational Research in Population Health (ITR), Neuroinova, University of Porto, Vila Nova de Gaia, Portugal; 4 Unidade Local de Saúde de Matosinhos, EPIUnit - Institute of Public Health, Laboratory for Integrative and Translational Research in Population Health (ITR), University of Porto, Porto, Portugal; 5 Institute of Electronics and Informatics Engineering of Aveiro, Medical Sciences Department, University of Aveiro, Aveiro, Portugal

**Keywords:** Information technology, Computer science

## Abstract

To explore the association between the user’s cognitive function and usability reported by the evaluator. A cross-sectional study was conducted with a community-based sample. Data about participants’ age, sex, education, sleep quantity, subjective memory complaints, and cognitive function were collected. A usability session was conducted to evaluate a digital solution called Brain on Track. Independent linear-regression analyses were used to explore univariable and multivariable associations between evaluator-reported usability assessment and the users’ cognitive function, age, sex, education, sleep quantity, and subjective memory complaints. A total of 238 participants entered this study, of which 161 (67.6%) were females and the mean age was 42 (SD 12.9) years old. All variables (age, education, sleep quantity, subjective memory complaints and cognitive function) except sex were significantly associated with evaluator-reported usability in the univariable analysis (p < 0.05). Cognitive function, age, education, and subjective memory complaints remained significant in the multivariable model (F = 38.87, p < 0.001) with an adjusted R2 of 0.391. Cognition scores alone showed an adjusted R^2^ of 0.288. This work suggests that cognitive function impacts evaluator reported usability, alongside other users’ characteristics and needs to be considered in the usability evaluation.

## Introduction

The ISO 9241-11 standard defines usability as the measure by which a product can be used by specific users to achieve specific goals with effectiveness, efficiency and satisfaction, in a context of specific use^1^. Therefore, usability is dependent on the context of use, i.e., the extent to which a system is considered usable depends on the specific circumstances in which the digital technology is being used^1^. The context of use includes users (and their characteristics), tasks being performed with the digital solution, equipment (hardware and software), as well as the physical and social environment where the digital solution is being used^1^.

Usability evaluation is a critical part of the development process of any digital solution, and must be considered to obtain a fully functional system, improve acceptability, increase user satisfaction and improve the digital solution’s reliability^2,3^. Usability evaluation can take place at any point in a digital solution lifecycle and should involve real users and usability experts throughout the process. For both of these, there is a variety of methods available that should be applied rigorously to collect accurate usability data that enables designers and developers to improve the digital solution^4–6^.

Despite the general assumption that users’ characteristics impact the results of usability evaluation, there is a lack of in-depth knowledge on which specific characteristics should be considered^7^. Users’ characteristics that are reported in the literature as conditioning the interaction with digital solutions include the user’s age^8–11^, education^9^, digital literacy^9,12–15^ and physical ability^16^. Another user characteristic likely to affect usability is the cognitive function, including the ability to understand, learn, be attentive, memorise, or be concentrated^8^. All these are required when interacting with any digital solution^17,18^ and, therefore, are likely to affect the ability and the experience of using a digital solution. To the best of our knowledge, no study has previously explored the association between cognitive function and usability in cognitively unimpaired individuals. However, previous studies using individuals with mild cognitive impairment and dementia have shown that usability measures were poorer in these individuals (they complete fewer tasks, take longer to complete them or need more help) when compared with older adults without cognitive impairment^19,20^.

These findings suggest that cognitive functioning might influence evaluators perceived usability, which is based on the performance of the individual while interacting with the digital solution during the usability evaluation. Therefore, this study aims to explore the association between cognitive function and usability reported by the evaluator in a community of cognitively unimpaired dwelling adults.

## Methods

This is a cross sectional study approved by the Ethics Committee of the *Centro Hospitalar de Entre o Douro e Vouga, EPE*, Portugal (process number CA-0114/16-0c, date 25 Jan 2016). All participants signed an informed consent prior to entering the study and all methods were performed in accordance with the relevant guidelines and regulations.

### Participants and inclusion and exclusion criteria

A community-based sample of residents of the region of Águeda, Aveiro, Portugal was involved in the study. To recruit participants, the study was publicized in the local media, in local dissemination actions and through leaflets placed at public establishments. Also, institutions linked to the public administration, industry, education, and the social area of the municipality were contacted, and employees and customers were invited to participate in the study. To be included, participants had to be 18 years old or older, be able to use a computer independently and be able to read in Portuguese. Participants were excluded if having cognitive deficit as assessed by the Montreal Cognitive Assessment (MoCA) as described below in the Procedures section.

### Procedures

Participants who agreed to enter the study were asked to provide data regarding age, sex, years of formal education, sleep quantity, subjective memory complaints, and cognitive function.

Sleep quantity was evaluated by self-reporting of the average hours of sleep per night on the last month.

Subjective memory complaints were assessed using the Subjective Memory Complaints Scale (SMC). The SMC consists of ten items^21^. Each individual item is scored zero (in case of absence of complaints), one, two or three points (in case of maximum complaints) depending on the severity of the complaint. Total score varies between zero and of 21 points. The European Portuguese version of this scale is valid and reliable^22^. Values greater than or equal to four points indicate significant memory complaints and values less than or equal to three indicate no relevant memory complaints^22^.

Cognitive function was assessed with the MoCA, which evaluates different cognitive domains, namely executive function; visuospatial capacity; memory; attention, concentration and working memory; language and temporal and spatial orientation. It has been shown to have high sensitivity (90%) and specificity (87%) for detecting mild cognitive impairment^23^. It is validated for the Portuguese population^24^ and total score ranges from 0 to 30 points, with higher scores representing better cognitive performances^23^. The cut off points for cognitive deficit vary between 16 and 27, depending on the participants age and education level, as defined by the Portuguese normative data^25^. According to the normative data for the Portuguese population, the MoCA scores are expressed as the means ± standard deviation (SD), and those of the distributions that are below 1 SD, 1.5 SDs, and 2 SDs can be considered as cutoff points for possible cognitive impairment^25^. To determine the MoCA cutoff points, the age and education are considered, and the SD used for this study was 1.5. MoCA was selected because it is a highly sensitive tool for early detection of mild cognitive impairment (MCI) and is widely used in research and clinical practice. Besides that, MoCA is validated for Portugal and has normative data for the Portuguese population. MoCA was used both to screen for inclusion and to characterize the cognitive function of participants who entered the study.

### Usability evaluation

Usability evaluation took place in a single session. Participants were asked to use a digital solution called Brain on Track. This digital solution is a computerized web-based self-administered test intended for cognitive testing that allows monitoring of the cognitive performance of users^20,21^. This solution has gone through a demanding process of testing and validation and is on the market since 2013.

The usability session consisted of three parts:

1. Pre-test—The evaluator explained all the procedures of the study.
2. Test—The evaluator explained how to use the digital solution Brain on Track and the participant interacted freely with it for 25 min. The evaluator helped the participant whenever the participant had doubts about any feature of the system and observed the interaction.
3. Post-test—The evaluator filled the ICF based Usability Scale I (ICF-US I)^26^. The ICF-US I is a usability rating scale that can be used as a tool for the usability evaluation based on the evaluator’s opinion^26^. This scale was used in this study because usability based on the evaluator’s perspective showed higher correlation with indicators of user performance than usability based on the user’s perspective^2^. The ICF-US I is composed of 10 items associated with different usability principles that are classified as facilitators or barriers. It is based on the International Classification of Functioning, Disability and Health (ICF)^27^ classification of environmental factors^28^. Each item is scored from − 3 (barrier) to 3 (facilitator) or not applicable (NA). For purposes of scoring, if an item is classified as NA, then this item score will be replaced by the mean value of the remaining items, rounded to the units. The final score of the ICF-US I is calculated by adding the scores of all individual items up to a maximum score of 30. Values above 10 indicate good usability and values below 10 indicate poor usability. A total of four evaluators with at least 2 years of experience in usability evaluation participated in the study, but each participant was observed by one evaluator only.

### Statistical analysis

All data analyses were performed using SPSS 28.0 for Windows (SPSS Inc, Chicago, IL). Mean and standard deviation and count and proportion were used to describe continuous and categorical variables, respectively. To determine possible factors associated with evaluator reported usability, independent linear-regression analyses were used to explore univariable and multivariable associations between the independent variables (age, sex, education, sleep quantity, subjective memory complaints and MoCA scores) and usability based on the opinion of the evaluator as the dependent variable. Normality was checked by visual inspection of the plots and no major violations were noted for the independent variables. The univariable analyses was used to identify the variables that would enter the multivariable model, and p ≤ 0.10 was required, except for sex and age, which were forced into the multivariable model. The multivariable analyses were performed using the enter, stepwise, backward and forward models and as the Adjusted R-squared of the models varied less than 0.01, the results of the stepwise model were reported. The variables that entered into the models were checked for multicollinearity using variance inflation factor (VIF) ≤ 5 and the respective tolerance value (> 0.2). Level of significance was set at p < 0.05.

## Results

A total of 238 participants entered this study, of which 161 (67.6%) were females. The mean age was 42 (SD 12.9) years old (range 20–76 years old). Sample characteristics are presented in Table 1.

**Table 1:** Sample characteristics (n = 238).

Variables	
Sex, n (%)	
Female	161 (67.6)
Male	77 (32.4)
Age, mean (± SD)	42 (± 12.9)
Years of formal education, mean (± SD)	14 (± 3.9)
Hours of sleep, mean (± SD)	6.8 (± 1.1)
SMC total (0–21), mean (± SD)	3.9 (± 2.8)
MoCA total (0–30), mean (± SD)	27.17 (± 2.1)

The mean (± SD) score of the ICF-US I was 25.38 (± 6.89), which indicates that the digital solution Brain on Track was considered a big facilitator. Of the 238 participants, 230 (96.6%) scored above the cut off (10 points) for considering the application a facilitator. The remaining 8 participants scored the digital solution below the cut off indicating that the application was considered a barrier.

### Univariable associations with evaluator reported usability

All variables (education, sleep quantity, subjective memory complaints and cognitive function) were significantly associated with usability (Table 2) in the univariable analysis.

**Table 2:** Univariable associations with evaluator-reported usability.

Independent variables	Unstandardized coefficient (95% CI)	Standardized coefficient	p
Education (years)	0.85 (0.64; 1.05)	0.47	< 0.001
Sleep quantity (hours)	1.21 (0.45; 1.97)	0.20	0.002
Subjective memory complaints	− 0.51 (− 0.81; − 0.20)	− 0.21	0.001
MoCA scores	1.77 (1.42; 2.12)	0.18	< 0.001

### Multivariable associations with evaluator reported usability

Education, sleep quantity, subjective memory complaints, MoCA, sex and age were all include in the multivariable analysis as independent variables. Of these, age, education, subjective memory complaints and cognitive function remained significant in the multivariable model (F = 38.87, p < 0.001) with an adjusted R^2^ of 0.391 (Table 3). MoCA scores alone showed an adjusted R^2^ of 0.288.

**Table 3:** Multivariable regression models.

Model	Unstandardized coefficient (95% CI)	Standardized coefficient	p
Constant	2.36 (− 9.67; 14.38)		
MoCA score	0.88 (0.44; 1.32)	0.27	< 0.001
Age	− 0.13 (− 0.19; − 0.07)	− 0.24	< 0.001
Education	0.42 (0.20; 0.64)	0.23	< 0.001
Subjective memory complaints	− 0.35 (− 0.60; − 0.10)	− 0.15	0.006

## Discussion

This study explored the association between cognitive function and usability reported by the evaluator in a community-based sample and results suggest that cognitive function impacts evaluator reported usability, alongside with age, education and subjective memory complaints.

Cognitive function, accounted for most of the variance of usability explained by the multivariable model, which in total explained around 39% of the usability variance. The remaining 61% may be explained by the intrinsic usability of the system, and other variables that are yet to be studied.

Studies have suggested that using digital solutions, such as personal computers^29^ or tablets^30^ help maintain or enhance cognitive functions, such as memory, processing speed and attention. We are not aware of previous studies exploring the association between cognitive function and the perceived usability of the study neither in the evaluator’s perspective nor in the user’s perspective. However, for a user to be efficient in the use of a certain digital solution, several cognitive functions are involved, including concentration, attention, and memory^31^. Conceivably, the better the user's ability in these areas, the easier the interaction with this type of technologies will be. On the other hand, those users with lower cognitive ability may have more difficulty interacting with complex digital solutions. The findings of the present study suggest that assessing users’ cognitive function in usability studies is relevant and raise important questions, namely to what extent is it necessary to differentiate the interfaces according to the cognitive skills of the users? Another important issue is the role of training, as the association between cognitive function and usability might decrease with continued use, but individuals with lower cognitive skills might require longer periods of training to be able to effectively and efficiently use a digital solution. Also, this study results reinforce the importance of applying usability principles in the design of digital solutions such as minimize memory load, meaning that users should be able to recognize rather than recall or use minimalist design to avoid distractions and favor concentration^32,33^.

It is important to note that this study did not include individuals with a previous diagnosis of mental or neurological diseases, but people living in the community with no identified cognitive impairment.

A limitation of the present study is the fact that digital literacy was not considered, and it is one of the variables mentioned in the literature as influencing usability^10,34^. This variable is difficult to assess comprehensively, as it is not just about being proficient in the use of technology, but also about its ethical and responsible use and it is related to social and cultural aspects^35^. Another study limitation is the fact that neither user self-perceived usability nor objective measures of usability were evaluated, and it would be interesting to explore whether they are also associated with cognitive function. The ICF-US I was used to study the association with usability reported by the evaluator as it is a reliable tool that overcomes the difficulty reported in the literature related to the fact that the opinion of the users, collected through the filling of self-perceived generic usability scales, do not fully reflect users’ performance^26,36^.

## Conclusion

Cognitive function seems to be associated to the usability of digital solutions and influence the human-technology interaction. The results of this study suggest that cognition has an even more pronounced influence in usability than other user characteristics such as age or education.

## Data Availability

All data needed to evaluate the conclusions in the paper are present in the paper. Additional data related to this paper may be requested from the authors.
